# From cancer safety signals to cardio-oncology translation: a dual-database bibliometric and BERTopic semantic analysis of SGLT2 inhibitor research in oncology (2014–2026)

**DOI:** 10.3389/fonc.2026.1969057

**Published:** 2026-10-21

**Authors:** Xianqin Tang, Wenlong Niu, Zhenxiong Lan, Zilan Zhong

**Affiliations:** 1 The First Clinical Medical College of Guangzhou University of Chinese Medicine, Guangzhou, Guangdong, China; 2 Shenzhen Clinical Medical College, Guangzhou University of Chinese Medicine, Shenzhen, Guangdong, China

**Keywords:** BERTopic, bibliometrics, cancer, cardio-oncology, drug repurposing, real-world evidence, SGLT2 inhibitors

## Abstract

**Objective:**

The clinical value of SGLT2 inhibitors has expanded from glycemic control to cardiorenal protection, while their oncology-related research encompasses long-term safety, tumor metabolism, real-world outcomes, and cardio-oncology. This study aimed to characterize the knowledge evolution of this field from cancer safety signals to testable clinical-translational questions and to distinguish research attention from evidence maturity.

**Methods:**

English-language records were retrieved from Web of Science Core Collection (WoSCC) and PubMed from January 1, 2012, to July 26, 2026. The final audited corpus comprised 346 records (258 Articles and 88 Reviews). Reviews were retained as a separate descriptive stratum. The full corpus was used for publication, controlled-concept, and exploratory evidence-map analyses, whereas Article records were used for the primary BERTopic model and Article-specific inferential analyses. The WoSCC subset (n=283) was used for citation and co-citation analyses. Annual Article counts were modeled using Poisson regression with annual PubMed English-language journal-article volume as an offset to account for background growth in biomedical publishing; a sensitivity BERTopic model additionally included Reviews.

**Results:**

A total of 346 records were included, of which 283 entered the citation analysis. Full-corpus publication output increased from 4 records in 2014 to 101 in 2025, while Article output increased from 3 to 67. After adjustment using annual PubMed English-language journal-article volume as the reference, the PubMed-referenced Article activity index showed an estimated annual IRR of 1.35 (95% CI, 1.28–1.43; P<0.001). Article-only BERTopic assigned 222 of 258 records to 10 topics and retained 36 outliers. The Article+Review sensitivity model yielded 13 topics with ARI = 0.634 and AMI = 0.698 after centroid alignment.

**Conclusions:**

SGLT2 inhibitor oncology research has expanded into parallel tracks of cancer safety, mechanistic antitumor hypotheses, real-world outcomes, and cardio-oncology supportive care. The bibliometric and semantic results describe indexed research attention rather than treatment efficacy or causal benefit. Available clinical evidence remains heterogeneous, and no overall class-wide cancer-risk increase has been confirmed without excluding rare, site-specific, agent-specific, or latency-related uncertainty. Prospective and randomized studies should address drug specificity, clinically achievable exposure, cancer-specific endpoints, treatment continuity, and patient-relevant outcomes.

## 1 Introduction

Cancer, type 2 diabetes, and cardiovascular disease frequently coexist in the same patients, and the increasing global cancer burden means that evaluations of glucose-lowering agents can no longer be limited to glycemic control (1). SGLT2 inhibitors have demonstrated consistent benefits in studies of heart failure and kidney outcomes (2, 3). As the eligible population expands, situations in which patients with cancer receive these agents before, during, or after treatment are becoming increasingly common.

Previous research has broadly followed three paths. First, randomized trials and their evidence syntheses have mainly evaluated overall and site-specific cancer risks; existing meta-analyses have not indicated a clear overall risk increase, although rare events, the latency of follow-up, and between-drug differences still limit certainty (4, 5). Second, preclinical studies have proposed candidate mechanisms involving glucose transport, mitochondrial respiration, AMPK–mTOR signaling, oxidative stress, apoptosis, and immune modulation (6). Third, the cardiovascular benefits of SGLT2 inhibitors have prompted research on cancer therapy-related myocardial injury, with emerging signals from prospective pilot studies (7). Current cardio-oncology guidelines emphasize risk stratification, dynamic monitoring, and multidisciplinary management (8).

Recent research has further advanced these questions to the level of clinical outcomes. Drug-target Mendelian randomization and multi-source observational analyses have suggested a signal for prostate cancer risk (9); sequential target-trial emulation has begun to evaluate treatment failure in the context of hormone therapy (10); active-comparator cohorts have extended the analysis to cancer-specific outcomes, including hepatocellular carcinoma (11). These studies do not constitute homogeneous evidence: mechanistic studies address whether an effect is possible, observational studies address whether an association exists in specific populations, and intervention studies directly test clinical benefit. Knowledge evolution and evidence stratification should therefore be interpreted within the same framework.

This study integrates dual-database bibliometrics, PubMedBERT-BERTopic semantic topic modeling, and an exploratory translational evidence map to summarize research growth and collaboration patterns, reconstruct the knowledge bases of journals and cited references, and identify fine-grained topics and their temporal trajectories in a data-driven manner. Because citation indicators reflect scholarly attention rather than evidence quality, we further present an evidence gradient from safety signals to clinical translation by drug, cancer context, research role, and evidence level. The overall writing structure was informed by recent paradigms that combine multi-database bibliometrics with clinical evidence synthesis (12, 13).

## 2 Materials and methods

### 2.1 Study design, data sources, eligibility, and search strategy

This bibliometric and semantic topic-modeling study was based on publicly available bibliographic metadata and did not involve patient-level interventions or effect-size pooling. We systematically searched WoSCC and PubMed, each from January 1, 2012, to July 26, 2026. The final search was completed on July 26, 2026. As the first eligible publication appeared in 2014, temporal trends are presented for 2014–2026. 2026 is an incomplete year ending on the search date. WoSCC was limited to English-language Articles or Reviews, and PubMed records were screened using the same oncology-relevance and language criteria. Publication type was adjudicated after deduplication using database metadata plus author review of records that lacked an explicit type field.

To accommodate differences in database fields, WoSCC used the Topic field (TS), whereas PubMed combined MeSH terms with Title/Abstract free-text terms. The WoSCC search query was: TS=((“SGLT2 inhibitor*” OR “SGLT-2 inhibitor*” OR SGLT2i OR SGLT-2i OR “Sodium-Glucose Cotransporter 2 inhibitor*” OR “Sodium Glucose Cotransporter 2 inhibitor*” OR “Sodium-Glucose Transporter 2 inhibitor*” OR “Sodium Glucose Transporter 2 inhibitor*” OR Gliflozin* OR Canagliflozin* OR Dapagliflozin* OR Empagliflozin* OR Ertugliflozin* OR Ipragliflozin* OR Luseogliflozin* OR Tofogliflozin* OR Sotagliflozin* OR Bexagliflozin* OR Remogliflozin* OR Sergliflozin* OR Licogliflozin*) AND (cancer* OR tumour* OR tumor* OR neoplasm* OR malignan* OR oncolog* OR carcinoma* OR adenocarcinoma* OR sarcoma* OR melanoma* OR leukemia* OR leukaemia* OR lymphoma* OR metastat*)). This search retrieved 1,019 records.

The PubMed search query was: (“Sodium-Glucose Transporter 2 Inhibitors”[Mesh] OR “SGLT2 inhibitor*”[Title/Abstract] OR “SGLT-2 inhibitor*”[Title/Abstract] OR SGLT2i[Title/Abstract] OR SGLT-2i[Title/Abstract] OR “Sodium-Glucose Cotransporter 2 inhibitor*”[Title/Abstract] OR “Sodium Glucose Cotransporter 2 inhibitor*”[Title/Abstract] OR “Sodium-Glucose Transporter 2 inhibitor*”[Title/Abstract] OR “Sodium Glucose Transporter 2 inhibitor*”[Title/Abstract] OR Gliflozin*[Title/Abstract] OR Canagliflozin*[Title/Abstract] OR Dapagliflozin*[Title/Abstract] OR Empagliflozin*[Title/Abstract] OR Ertugliflozin*[Title/Abstract] OR Ipragliflozin*[Title/Abstract] OR Luseogliflozin*[Title/Abstract] OR Tofogliflozin*[Title/Abstract] OR Sotagliflozin*[Title/Abstract] OR Bexagliflozin*[Title/Abstract] OR Remogliflozin*[Title/Abstract] OR Sergliflozin*[Title/Abstract] OR Licogliflozin*[Title/Abstract]) AND (“Neoplasms”[Mesh] OR Cancer*[Title/Abstract] OR Tumor*[Title/Abstract] OR Tumour*[Title/Abstract] OR Neoplasm*[Title/Abstract] OR Malignan*[Title/Abstract] OR Oncolog*[Title/Abstract] OR Carcinoma*[Title/Abstract] OR Adenocarcinoma*[Title/Abstract] OR Sarcoma*[Title/Abstract] OR Melanoma*[Title/Abstract] OR Leukemia*[Title/Abstract] OR Leukaemia*[Title/Abstract] OR Lymphoma*[Title/Abstract] OR Metastat*[Title/Abstract]). This search retrieved 1,184 records.

All records (n=2,203) were imported into EndNote 2025. Duplicates were first matched by DOI or PMID and then manually checked by title, author, and year. Eligible records were English-language articles and reviews with a substantive association with cancer risk, cancer incidence or mortality, cancer biology, antitumor drug repurposing, cancer-care outcomes, or the prevention and management of treatment-related toxicities involving SGLT2 inhibitors/SGLT2/SLC5A2. Duplicate records, preprints, records with ineligible publication types, and publications lacking a substantive oncology question were excluded. After removal of 854 duplicates, 1,349 unique records were screened. Of these, 1,003 were excluded, yielding a final sample of 346 publications. Record-level adjudication resulted in 258 Articles and 88 Reviews. The 12 records initially flagged as Other were author-adjudicated as Reviews. Retrieval and eligibility screening were cross-checked by two researchers, with disagreements resolved by discussion with a third reviewer. Independent double-coding was applied specifically to the exploratory **Figure 2** labels using the prespecified codebook and validation procedure described separately.

**Figure 1:**
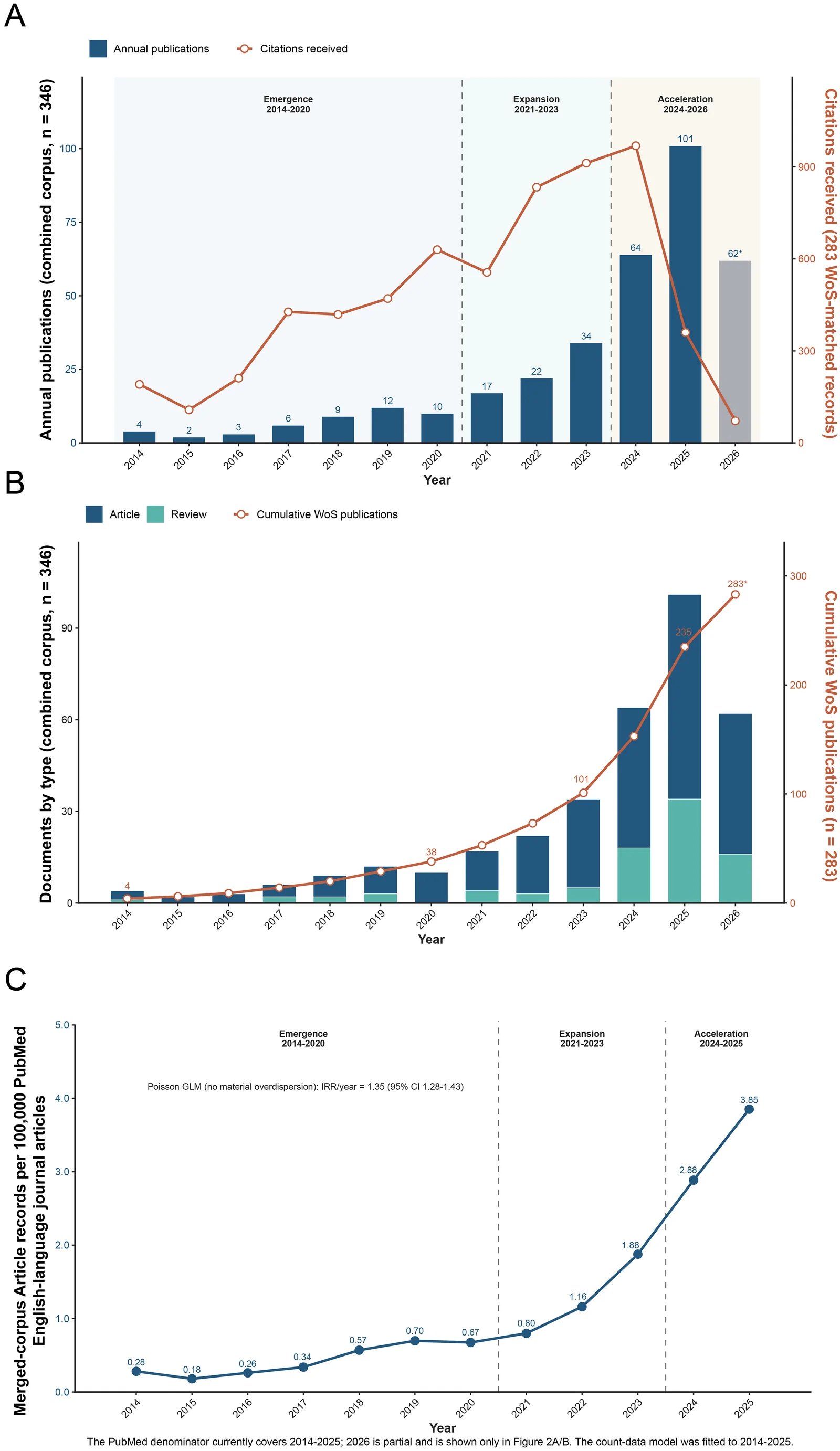
Publication output, document-type composition, and PubMed-referenced Article publication activity, 2014–2026. **(A)** Annual publications in the full combined WoSCC–PubMed corpus and citations received by the 283 matched WoS records; the 2026 bar is partial. **(B)** Annual Article and Review records in the combined corpus, with cumulative publications in the WoS subset shown on the secondary axis. **(C)** PubMed-referenced Article publication activity index, defined as the number of audit-confirmed Article records from the deduplicated WoSCC–PubMed merged corpus per 100,000 English-language journal articles indexed in PubMed in the same year. Each record was counted once regardless of whether it was indexed in PubMed, WoSCC, or both. The PubMed series was used as the reference denominator and Poisson offset. Points show observed index values for 2014–2025; the count-data model used a Poisson generalized linear model with the log annual PubMed count as an offset. The incomplete 2026 corpus is not included in the index or model inference.

**Figure 2:**
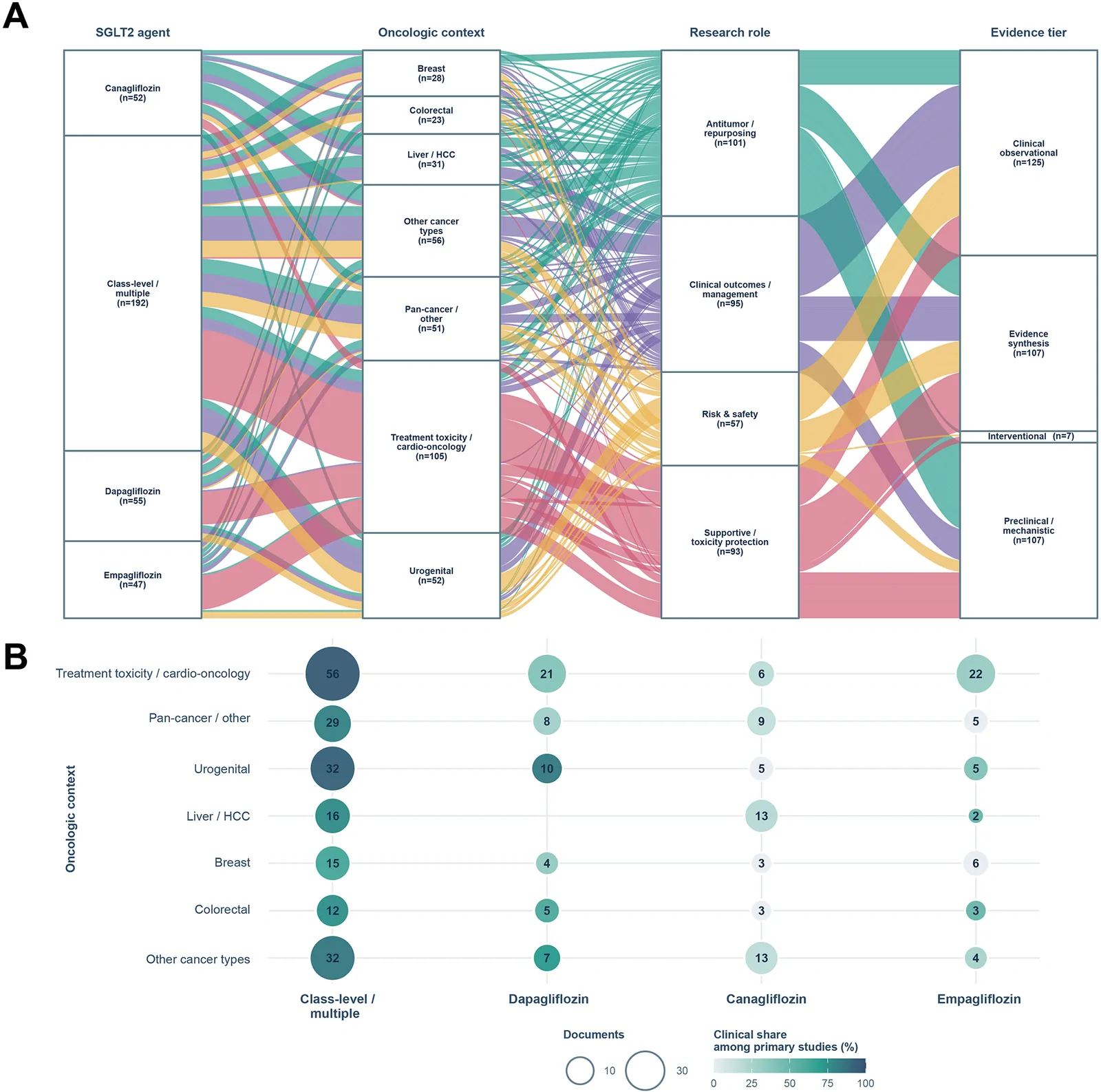
Exploratory landscape of translational evidence. **(A)** Four-axis alluvial plot linking SGLT2 agents, oncologic contexts, research roles, and evidence tiers; flow width is proportional to document count. **(B)** Drug–context bubble matrix; bubble size represents document count and fill represents the percentage of primary studies classified as clinical observational or interventional. Evidence syntheses, including all audit-confirmed Reviews, were excluded from this denominator. Two independent coders classified all 346 records; Cohen’s κ was 1.000 for agent, 0.972 for context, 0.965 for research role, and 0.987 for evidence tier. “Class-level/multiple” indicates class-level, multiple-agent, other-gliflozin, or non-unique bibliographic scope and does not represent patient-level co-exposure.

The identification and screening process followed the PRISMA 2020 flow diagram and PRISMA-S reporting items (**Figure 3**) (14, 15). To avoid misinterpreting database-coverage differences as differences in knowledge structure, the merged WoSCC–PubMed corpus (n=346) was used for descriptive, controlled-concept, BERTopic, and exploratory translational analyses. The 283 publications matched to complete WoSCC citation metadata constituted the citation subset for citation, journal knowledge-flow, and co-citation analyses.

**Figure 3:**
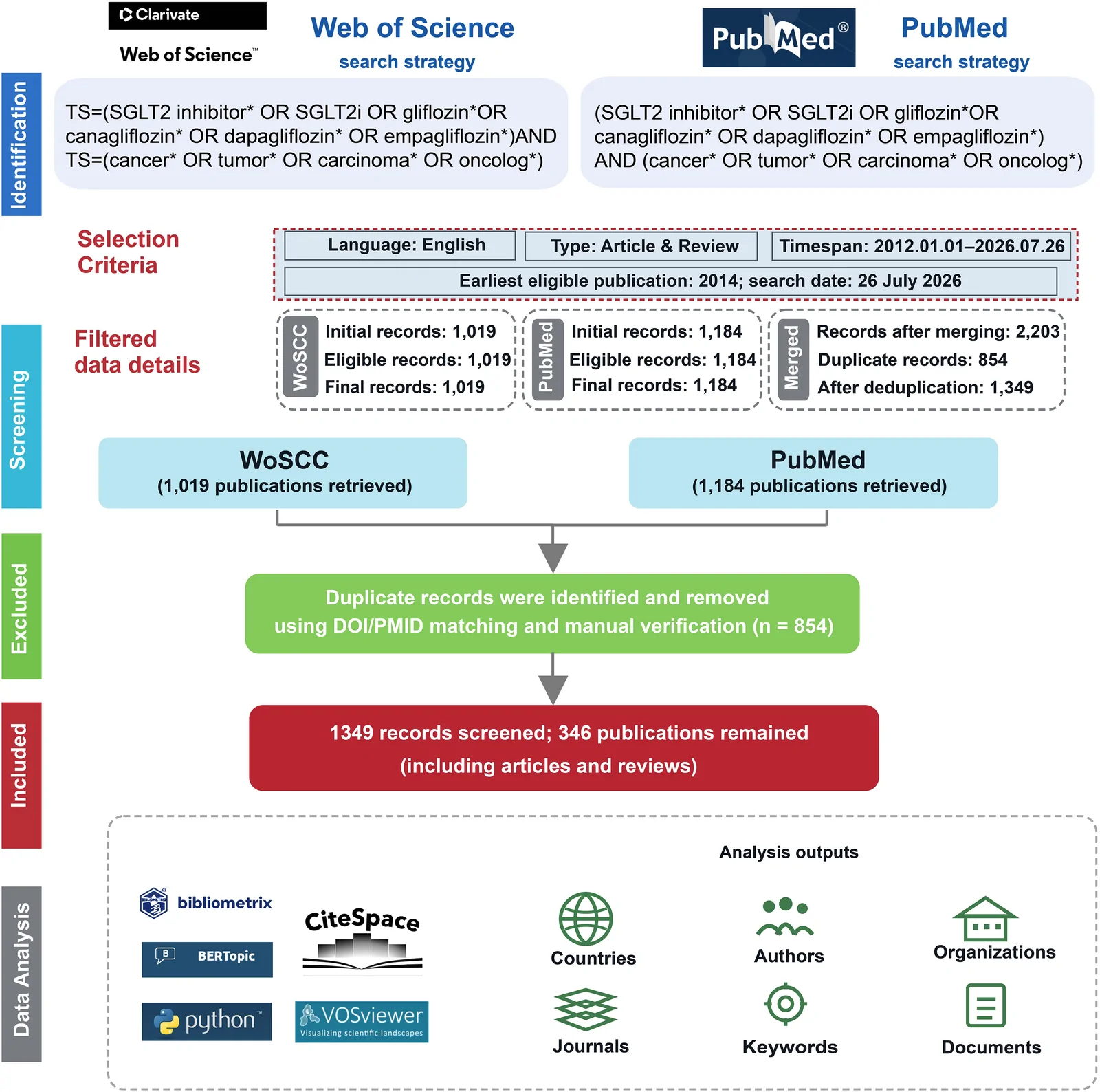
Workflow for literature identification, deduplication, screening, and construction of the analytical corpus. A total of 2,203 records were retrieved from WoSCC and PubMed; after removal of 854 duplicates, 1,349 unique records were screened and 346 publications were ultimately included. The merged corpus was used for descriptive, controlled-concept, BERTopic, and exploratory translational analyses; the matched 283 WoSCC records were used for citation, journal knowledge-flow, and co-citation analyses.

### 2.2 Bibliometric, controlled-concept, and BERTopic analyses

Annual output was tabulated for the full corpus and separately by document type. **Figure 1A** shows descriptive annual publication counts and the citation trajectory for the 283 WoS-matched records. **Figure 1B** shows Article and Review records together with cumulative WoS publications. **Figure 1C** expresses the annual number of audit-confirmed Article records in the deduplicated WoSCC–PubMed merged corpus per 100,000 English-language journal articles indexed in PubMed in the same year. Each merged-corpus record was counted once, regardless of whether it was indexed in PubMed, WoSCC, or both. The PubMed series served only as the reference denominator and as the offset for the Poisson model. The 2014–2025 Article counts were modeled with a Poisson generalized linear model using the log annual PubMed count as an offset because the Pearson dispersion diagnostic indicated no material overdispersion (dispersion=0.573). 2026 was excluded from inference for the PubMed-referenced activity index because the denominator series ends in 2025 and the corpus is incomplete. bibliometrix was used to calculate publication counts, citation counts, h-index, total link strength, and active years. Citation indicators describe scholarly visibility and do not replace study-quality or clinical-evidence assessments (16).

CiteSpace 6.4.R2 was used for cited-reference co-citation, cluster timelines, burst detection, and dual-map overlay of journals, whereas VOSviewer was used for author-keyword co-occurrence and average-publication-year overlay (17, 18). Networks used 1-year time slices, g-index node selection (k=25), and synonym cleaning. Source journals were also reported by productivity, citations, and Bradford-zone indicators.

Within the merged corpus of 346 publications, titles, abstracts, and keywords were classified into drug, cancer-site, mechanism, cardio-oncology, and evidence/translation concepts, and normalized frequencies were compared across 2014–2019, 2020–2023, and 2024–2026. A publication could be counted in more than one concept; 2026 data were accrued through July 26. **Figure 4D** reports concept frequencies rather than CiteSpace burst strength.

**Figure 4:**
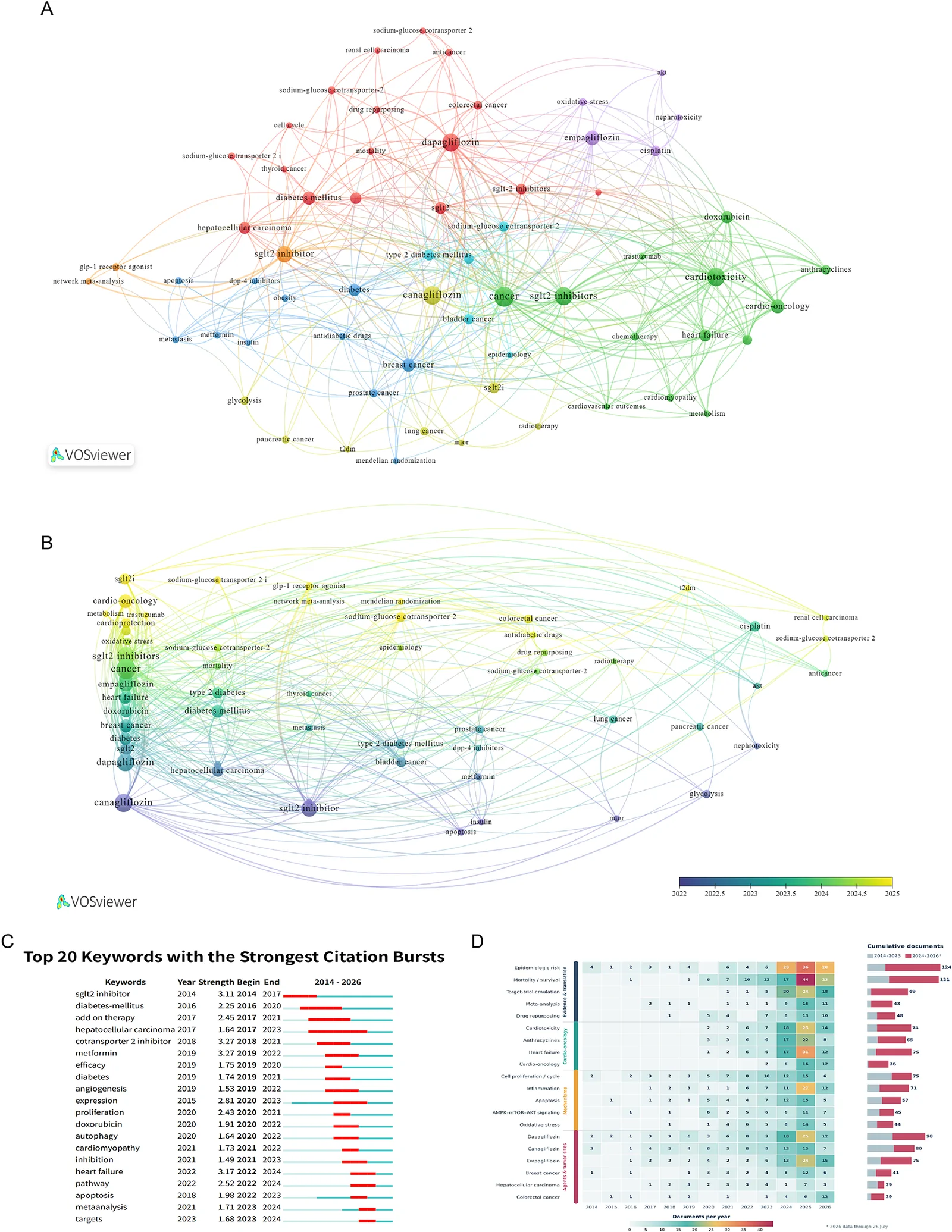
Keyword knowledge landscape and research frontiers. **(A)** Author-keyword co-occurrence network. Node size represents occurrence frequency, link width represents co-occurrence strength, and color represents cluster membership. **(B)** Average-publication-year overlay using the same network coordinates. **(C)** The 20 keywords with the strongest bursts; red segments indicate burst duration. **(D)** Annual-frequency heatmap and period composition of 20 controlled concepts in the merged corpus. Panel D reports transparent frequencies rather than burst strength.

BERTopic 0.17.4 was used for semantic topic identification (19) with PubMedBERT embeddings (NeuML/pubmedbert-base-embeddings). The primary model used the 258 Article records. A sensitivity model used all 346 records. Titles and abstracts were concatenated for each document, with titles alone used when abstracts were unavailable. PubMedBERT generated 768-dimensional embeddings, followed by UMAP (five dimensions) and HDBSCAN clustering. Topics were represented using class-based TF-IDF. A two-dimensional UMAP was used only for visualization in **Figure 5A**. Topics were aligned across models by cosine similarity of topic centroids, and stability was summarized with ARI, AMI, and mean matched-centroid cosine similarity on the Article subset. Annual topic counts were modeled with Poisson generalized linear models using annual Article-corpus size as an offset, HC0 robust standard errors, and Benjamini-Hochberg correction across topics. 2026 was retained descriptively but excluded from complete-year inference.

**Figure 5:**
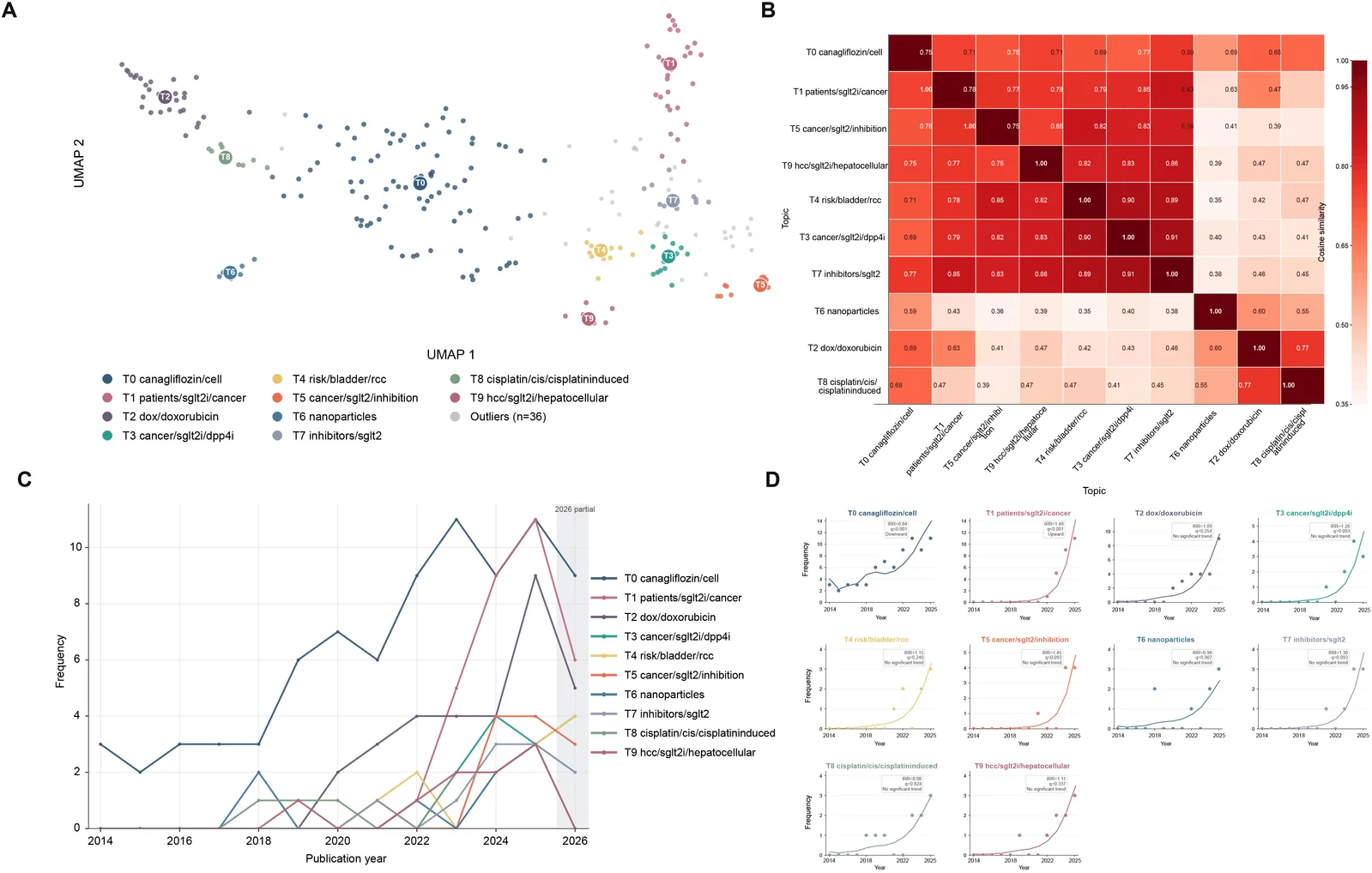
Article-only BERTopic structure and temporal trends. **(A)** UMAP projection of the 258 Article records, with 222 records assigned to 10 topics and 36 HDBSCAN outliers. **(B)** Clustered cosine-similarity matrix of Article-topic centroids. **(C)** Annual topic frequencies; 2026 is shown descriptively as a partial year. **(D)** Topic-specific Poisson GLM trends for complete years (2014–2025), using annual Article-corpus offsets, HC0 robust standard errors, and Benjamini–Hochberg-adjusted q values. The Article+Review sensitivity model identified 13 topics; cross-model stability was ARI = 0.634, AMI = 0.698, and mean matched-centroid cosine similarity=0.938. Topic labels are descriptive summaries of high-frequency terms and do not imply validated biological or clinical entities.

### 2.3 Exploratory translational mapping and reproducibility

The exploratory translational evidence map used all 346 publications as its parent set and assigned one primary label on four dimensions: SGLT2 agent, oncologic context, research role, and evidence tier. Each dimension was coded once per document using a prespecified title/abstract/indexed-keyword codebook. Records mentioning multiple agents, class-level exposure, other gliflozins, or a non-unique agent were coded as Class-level/multiple; this is a bibliographic scope label and does not represent simultaneous patient-level exposure. The final audit table contained 258 Articles and 88 Reviews; audit-confirmed Reviews were forced to the Evidence synthesis tier and excluded from the Panel B primary-study clinical-share denominator. Two coders independently coded all 346 records. Agreement was high (Cohen’s κ=1.000 for agent, 0.972 for context, 0.965 for research role, and 0.987 for evidence tier); 17 records with at least one discrepancy were adjudicated using PubMed/registry verification. The final labels were checked by the authors against the source records, and this limited methodological use is disclosed according to journal policy. **Figure 2A** presents the four-axis alluvial map and **Figure 2B** presents document volume and clinical share; the map is exploratory and does not estimate treatment effects or risk of bias.

Data processing and analyses were conducted in R and Python. bibliometrix, VOSviewer, CiteSpace, and BERTopic 0.17.4 were used for bibliometric, network, temporal, and semantic topic analyses.

## 3 Results

### 3.1 Rapid growth and changes in publication-type composition

In the full combined corpus, annual output increased from 4 records in 2014 to 101 in 2025 (**Figure 1A**). When stratified by document type, Article output increased from 3 to 67 and Review output from 1 to 34 over the same period. By July 26, 2026, the partial-year corpus contained 46 Articles and 16 Reviews; this year was not used for annual model inference. The mixed total therefore describes overall indexed publication activity and should not be interpreted as a one-to-one increase in original-study activity.

After adjustment using the annual volume of English-language journal articles indexed in PubMed as the reference denominator, the PubMed-referenced Article activity index increased from 0.28 records per 100,000 background articles in 2014 to 3.85 in 2025 (**Figure 1C**). A Poisson count model estimated an incidence-rate ratio of 1.35 per calendar year (95% CI, 1.28–1.43; nominal P = 1.48×10^−26^; Pearson dispersion=0.573). This index describes relative growth in merged-corpus Article activity after accounting for background PubMed volume. It does not measure treatment efficacy, causal benefit, or evidence maturity. The WoSCC citation subset comprised 283 publications, and citation accumulation remained strongly dependent on publication age.

### 3.2 Countries, institutions, and authors

China had the highest publication output, followed by the United States and Taiwan (China); the United States, the United Kingdom, Canada, and Saudi Arabia had higher proportions of international collaboration (**Figure 6A**). Publication output and international connectivity capture complementary dimensions of national research activity. Growth in the leading countries occurred mainly after 2022, and the collaboration network was linked by nodes including China, the United States, Taiwan (China), Italy, and the United Kingdom (**Figures 6B, C**).

**Figure 6:**
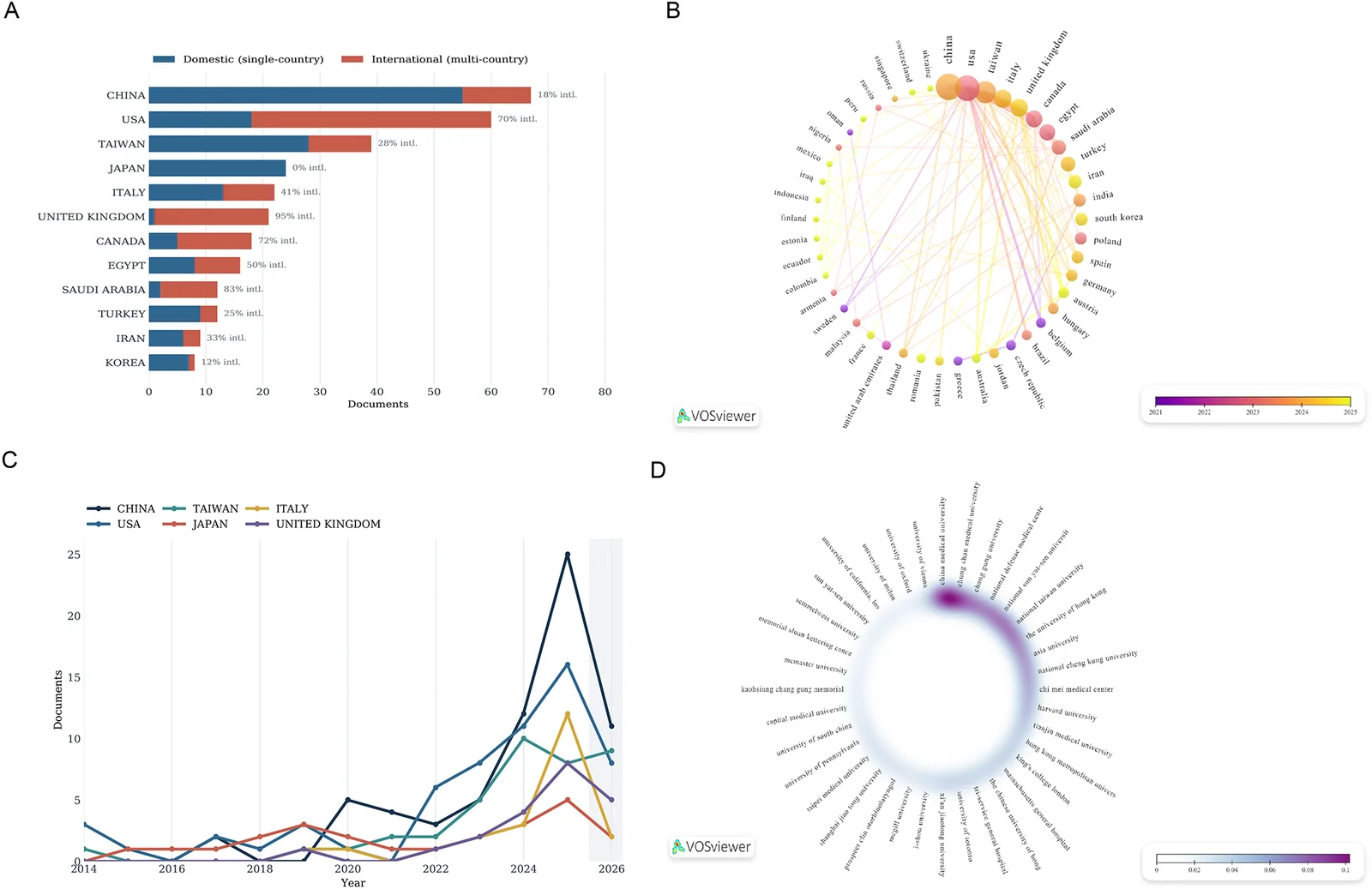
Contributions and collaboration patterns of countries and institutions. **(A)** Domestic and international collaboration composition for the 12 most productive countries/regions, with the proportion of international collaboration labeled. **(B)** Country-collaboration overlay map; node size represents publication output, link width represents collaboration strength, and color represents the average publication year. **(C)** Annual publication output for the six most productive countries/regions. **(D)** Institution-collaboration density map. The year 2026 is incomplete.

Institutional activity was concentrated primarily in research centers in East Asia and North America, with highly connected institutions also present in the United Kingdom (**Figure 6D**, **Table 1**). China Medical University ranked first with 15 publications, whereas Massachusetts General Hospital had the highest citation count, indicating high citation visibility in this field.

**Table 1:** Publication output and citation metrics of the 20 listed authors, ranked by total citations.

Rank	Institution	Documents	Citations	Avg. citations	Avg. pub. year
1	China Medical University	15	195	13	2024.3
2	Chung Shan Medical University	10	203	20.3	2024.1
3	Chang Gung University	9	44	4.9	2024.8
4	National Defense Medical Center	9	43	4.8	2025.1
5	National Sun Yat-sen University	9	56	6.2	2025.1
6	National Taiwan University	9	245	27.2	2023
7	The University of Hong Kong	9	146	16.2	2024.1
8	Asia University	8	55	6.9	2025.3
9	National Cheng Kung University	8	235	29.4	2023.5
10	Chi Mei Medical Center	7	156	22.3	2023.9
11	Harvard University	7	343	49	2023.9
12	Tianjin Medical University	7	134	19.1	2023.7
13	Hong Kong Metropolitan University	6	74	12.3	2024.3
14	King’s College London	6	39	6.5	2025.3
15	Massachusetts General Hospital	6	434	72.3	2023.3
16	The Chinese University of Hong Kong	6	132	22	2023.7
17	Tri-Service General Hospital	6	26	4.3	2025.3
18	University of Toronto	6	372	62	2023.2
19	Xi’an Jiaotong University	6	26	4.3	2024.2
20	I-Shou University	5	28	5.6	2025.4
21	McGill University	5	129	25.8	2022.8
22	Prospect Clin Otorhinolaryngol & Neurol	5	28	5.6	2025.4
23	Shanghai Jiao Tong University	5	80	16	2024.2
24	Taipei Medical University	5	115	23	2023.8
25	University of Pennsylvania	5	202	40.4	2024.6

**Table 2** summarizes publication output and citation metrics for 20 authors, ranked by total citations. Among the listed authors, Tomas G. Neilan had the highest publication count, with six papers and 434 citations. Paaladinesh Thavendiranathan and Anju Nohria each contributed four papers, receiving 308 and 289 citations, respectively. Philippe Armand contributed two papers with 182 citations, ranking fourth by total citations. Six other authors each contributed three papers.

**Table 2:** Top 20 most productive authors.

Rank	Authors	Documents	Citations	Avg. citations	Avg. pub. year
1	Tomas G. Neilan	6	434	72.3	2023.3
2	Paaladinesh Thavendiranathan	4	308	77.0	2023.0
3	Anju Nohria	4	289	72.3	2023.8
4	Philippe Armand	2	182	91.0	2022.5
5	Sourbha S. Dani	3	159	53.0	2024.3
6	Anita Deswal	3	159	53.0	2024.3
7	Sarju Ganatra	3	159	53.0	2024.3
8	Sumanth Khadke	3	159	53.0	2024.3
9	Ana Barac	3	157	52.3	2024.0
10	Eric H. Yang	3	157	52.3	2024.0
11	Bonnie Ky	2	150	75.0	2024.0
12	Jennifer Liu	2	150	75.0	2024.0
13	Marielle Scherrer-Crosbie	2	150	75.0	2024.0
14	Carlos A. Gongora	2	135	67.5	2023.5
15	Husam Abdel-Qadir	2	90	45.0	2023.5
16	Teresa Lopez-Fernandez	2	86	43.0	2025.0
17	Yu-Cheng Chang	2	77	38.5	2023.5
18	Cho-Han Chiang	2	77	38.5	2023.5
19	Cho-Hsien Chiang	2	77	38.5	2023.5
20	Cho-Hung Chiang	2	77	38.5	2023.5

### 3.3 Source journals and disciplinary knowledge flow

Source journals spanned oncology, pharmacology, endocrinology, and cardiovascular medicine (**Figure 7**). International Journal of Molecular Sciences ranked first with 12 publications, followed by Cancers and Scientific Reports with eight each; Biomedicine & Pharmacotherapy had fewer publications but a higher citation count than several higher-output journals, illustrating that publication output and citation impact were not fully concordant. Recent publications in JACC: CardioOncology reflect the growing prominence of the cardio-oncology theme.

**Figure 7:**
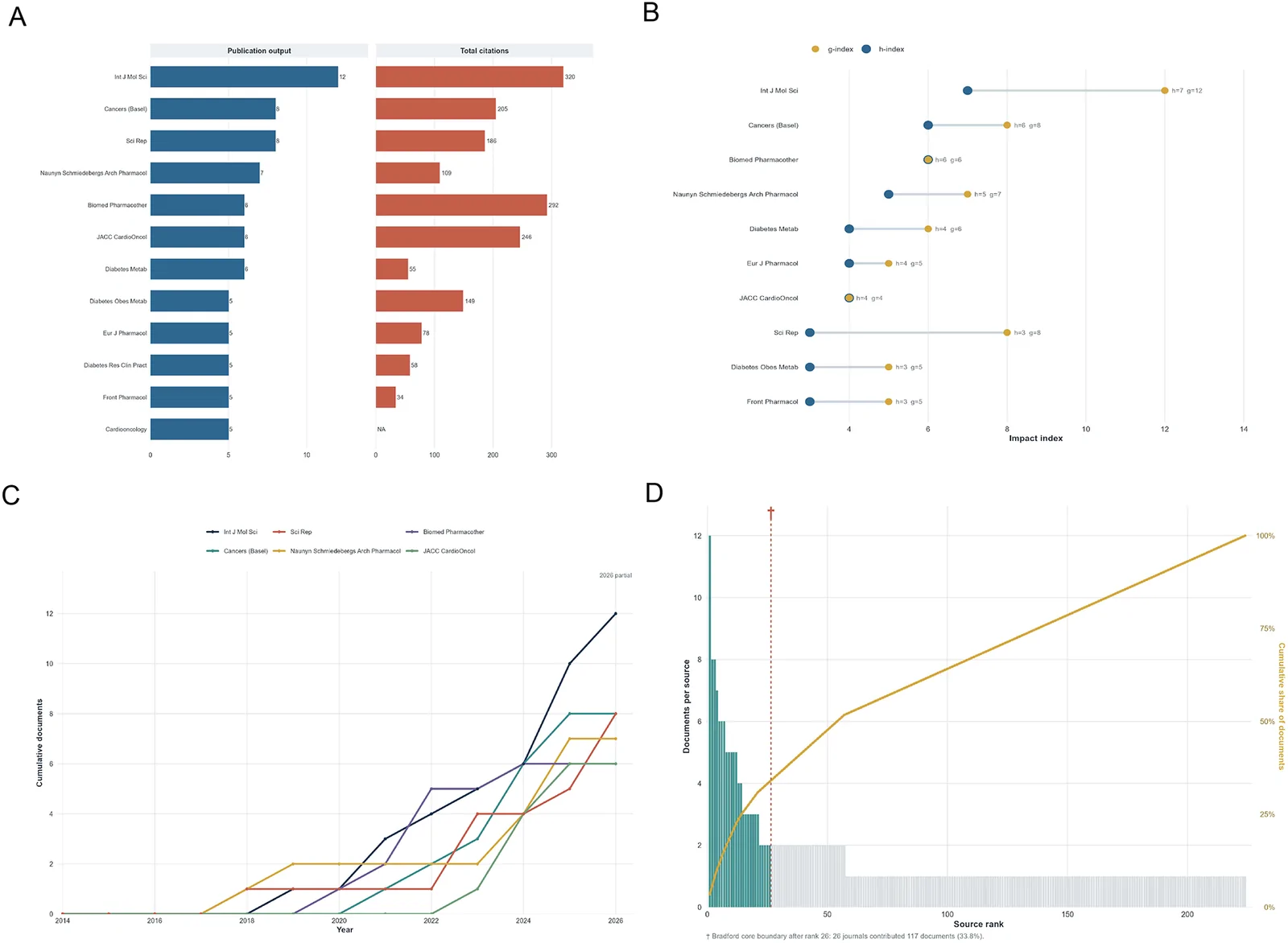
Source-journal landscape of the merged corpus (n=346). **(A)** Publication output and total citation counts of major source journals. **(B)** Local h-index and g-index. **(C)** Cumulative growth of six major journals. **(D)** Bradford distribution; the dashed line marks the core-zone boundary at rank 26, and the 26 core journals published 117 publications.

Early source journals focused more on pharmacology and mechanisms, whereas clinical and translational journal groups expanded markedly after 2023 (**Figure 7C**). The Bradford core zone comprised 26 journals and 117 publications (33.8% of the merged corpus); the remaining publications were distributed across a long journal tail, reflecting the interdisciplinary nature of this topic.

The dual-map overlay of journals showed that research fronts in molecular biology and immunology, as well as those in medicine and clinical research, primarily converged on a molecular/biology/genetics knowledge base (**Figure 8**). This suggests that experimental and clinical research fronts share a molecular knowledge base rather than forming mutually isolated disciplinary silos.

**Figure 8:**
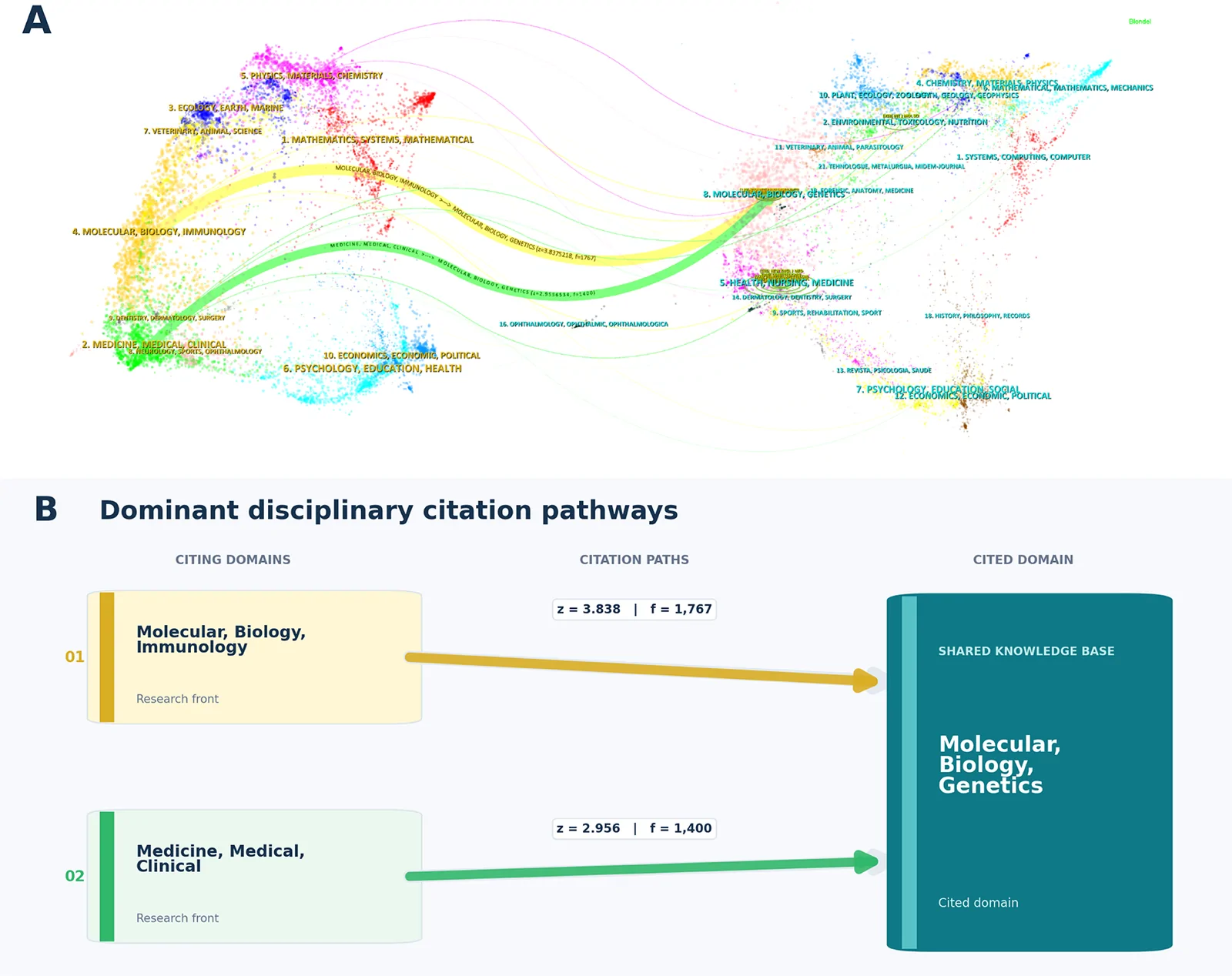
Dual-map overlay of journals and disciplinary knowledge flow. **(A)** Citing-journal disciplines are shown on the left and cited-journal disciplines on the right; colored curves represent major citation paths. **(B)** Quantitative summary of the two major paths. Molecular, biology, and immunology research fronts, as well as medicine and clinical research fronts, primarily cite a molecular/biology/genetics knowledge base. Path statistics were obtained using CiteSpace 6.4.R2.

### 3.4 Knowledge structure and co-citation evolution

The cited-reference co-citation network contained 511 nodes and 1,323 links, and its largest connected component comprised 342 references. The high modularity (Q = 0.81) and weighted mean silhouette value (S = 0.91) indicated a relatively clear and internally consistent cluster structure (**Figure 9A**). Eleven clusters formed a timeline extending from foundational safety and metabolic themes to recent translational research.

**Figure 9:**
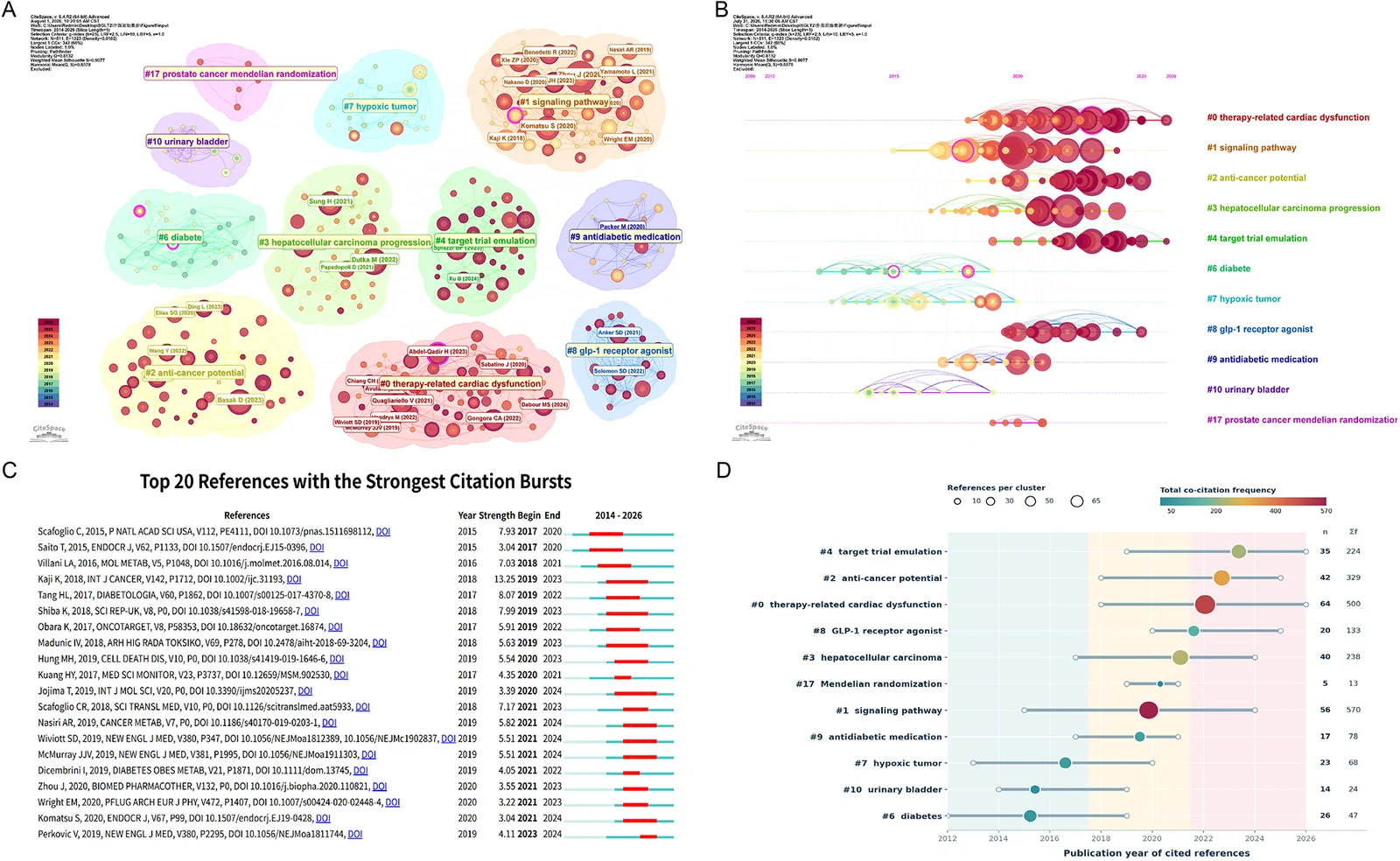
Knowledge structure and temporal evolution of co-cited references. **(A)** Cited-reference co-citation clustering network. Node size represents co-citation frequency, links represent co-citation relationships, and cluster labels were generated using the log-likelihood-ratio algorithm. **(B)** Timeline of major clusters. **(C)** References with the strongest citation bursts; red segments indicate burst intervals. **(D)** Cluster-characteristics plot integrating the time span of cited references, weighted mean year, number of references (bubble size), and total co-citation frequency (bubble color).

Historical clusters included diabetes, hypoxic tumors, bladder, and glucose-lowering agents; signaling pathways and hepatocellular carcinoma formed the mid-period knowledge base of mechanistic research. Clusters remaining active through 2025–2026 included cancer therapy-related cardiac dysfunction, antitumor potential, and target-trial emulation (**Figures 9B, D**).

Citation bursts gradually shifted from early cancer metabolism, hepatocellular carcinoma models, and the safety of glucose-lowering agents toward cardiovascular outcomes and cancer therapy-related cardiotoxicity (**Figure 9C**). Kaji et al. (20) showed the strongest citation burst among the references listed in **Figure 9C**. Evidence syntheses of cardiorenal outcomes formed an important background for recent clinical translation; anthracycline-treatment cohorts and prospective pilot studies further shifted research toward cancer therapy-related cardiotoxicity.

### 3.5 Keyword landscape and research frontiers

Seventy author-keyword nodes formed multiple interconnected thematic communities (**Figure 4A**). The central network linked SGLT2 inhibitors, cancer, cardiotoxicity, heart failure, doxorubicin, anthracyclines, and cardio-oncology; other communities highlighted diabetes and cancer metabolism, canagliflozin and metabolic signaling, dapagliflozin and risk terms, and empagliflozin with oxidative stress and cisplatin, respectively. The network contained both drug-specific mechanistic communities and cross-drug supportive-care communities.

The average-publication-year overlay showed that cardioprotection, cardio-oncology, drug repurposing, network meta-analysis, and target-trial emulation were more recent terms, whereas canagliflozin, glycolysis, mTOR, apoptosis, and several cancer-metabolism concepts were relatively earlier (**Figure 4B**). Overall, research has gradually shifted from single-drug mechanisms toward comparative and clinically oriented designs.

Keyword bursts shifted from SGLT2 inhibitor, diabetes, adjunctive therapy, and hepatocellular carcinoma toward heart failure, signaling pathways, apoptosis, meta-analysis, and target. Controlled-concept analysis showed that epidemiologic risk and mortality/survival were the concepts with the highest cumulative frequencies; Target-trial emulation and Cardiotoxicity were concentrated primarily in 2024–2026 and constitute the current frontiers (**Figures 4C, D**).

### 3.6 Fine-grained BERTopic topics and temporal trajectories

In the Article-only corpus, BERTopic assigned 222 of 258 records (86.0%) to 10 topics and retained 36 records (14.0%) as HDBSCAN outliers (**Figures 5A–C**). The largest topic was T0, characterized by canagliflozin/cell terms (82 assigned records), followed by T1, characterized by patients/sglt2i/cancer terms (32), and T2, characterized by dox/doxorubicin terms (31). The remaining topics covered cancer/sglt2i/dpp4i, risk/bladder/RCC, cancer/sglt2/inhibition, nanoparticles, inhibitors/sglt2, cisplatin-related terms, and HCC/sglt2i terms.

In topic-specific Poisson models fitted to complete years (2014–2025), only T0 and T1 retained statistically significant trends (**Figure 5D**). These IRRs describe changes in indexed Article-topic frequency after accounting for annual Article-corpus size.

The Article+Review sensitivity model identified 13 topics. After centroid-based alignment, Article assignments showed ARI = 0.634, AMI = 0.698, and a mean matched-centroid cosine similarity of 0.938, indicating substantial but not perfect stability after Reviews were added. Topic labels are descriptive summaries of high-frequency terms and are not validated biological or clinical entities.

### 3.7 Exploratory landscape of translational evidence

Using the prespecified codebook and final double-coding adjudication, the exploratory four-axis map displays all 346 records according to SGLT2 agent, oncologic context, research role, and evidence tier (**Figure 2A**). Treatment toxicity/cardio-oncology and pan-cancer/other were the predominant contexts, alongside urogenital, liver/hepatocellular, breast, colorectal, and other cancer types.

Antitumor/repurposing was the largest research-role category (101 records), followed by clinical outcomes/management (95), supportive/toxicity protection (93), and risk/safety (57). Evidence tiers comprised 125 clinical observational records, 107 preclinical/mechanistic records, 107 evidence syntheses, and 7 clinical interventional records. All 88 audit-confirmed Reviews were assigned to Evidence synthesis. Two independent coders agreed on 329 of 346 records across all four dimensions; Cohen’s κ was 1.000 for agent, 0.972 for context, 0.965 for research role, and 0.987 for evidence tier. Seventeen records with at least one disagreement were adjudicated using PubMed/registry verification, as documented in the final coding file.

In the drug–context matrix, bubble area represents document count and fill represents the clinical share among non-synthesis primary studies, to illustrate the research composition (**Figure 2B**).

## 4 Discussion: from research hotspots to translational evidence

### 4.1 Principal findings and evidence stratification

This study shows that indexed SGLT2 inhibitor oncology research has progressed from early safety concerns and metabolic mechanisms into parallel tracks of cancer risk, real-world outcomes, direct antitumor hypotheses, and cardio-oncology supportive care. Article-only BERTopic topics separated canagliflozin/cell, dox/doxorubicin, real-world patient/cancer terms, safety terms, nanoparticles, cisplatin-related terms, and hepatocellular-carcinoma terms. The field therefore appears to have expanded through parallel research tracks rather than a simple replacement of one topic by another.

The most important interpretive finding is that expanded research attention has not translated in parallel into clinical evidence of equivalent strength. Direct antitumor research remains dominated by preclinical and observational records (21), whereas cardio-oncology has progressed from mechanistic and observational work to selected prospective pilots (22, 23), and neither pattern establishes causal clinical benefit (24). The contextual clinical references discussed below were selected to support interpretation of the bibliometric findings. Accordingly, topic size and temporal momentum are interpreted as a research map, while study design, exposure attainability, and patient-relevant endpoints remain the bases for judging evidence maturity.

The dual-corpus design provides a methodological basis for this conclusion. The merged corpus of 346 publications retained PubMed’s biomedical coverage, whereas the WoSCC subset of 283 publications provided relatively harmonized citation and cited-reference fields. Their separate use avoids misreading differences in database coverage as differences in knowledge structure and permits a relatively clear distinction between research hotspots and knowledge impact.

### 4.2 Cancer risk: no overall signal of increase, but site-specific and single-drug uncertainty remains

Cancer safety is the starting point of this field and a necessary background for interpreting subsequent mechanistic and clinical research. Article-only BERTopic separated risk/bladder/RCC terms from recent patient/cancer terms, indicating a shift in research questions from early safety concerns toward comparative clinical inference. Available randomized-trial syntheses have not confirmed an overall class-wide increase in cancer risk (4); updated evidence syntheses likewise found no significant increase in the risk of bladder or breast cancer (5). Residual uncertainty for rare, site-specific, and agent-specific risks persists because of limited event counts and cancer-specific power, potentially insufficient follow-up for cancer latency, competing mortality, and between-drug differences (25). Historical agent-specific bladder-cancer imbalances discussed during early drug development, including canagliflozin-related safety concerns, remain a reason for continued surveillance; subsequent data have not established such signals as a class effect. Observational reports of lower hepatocellular or renal-cell carcinoma incidence are hypothesis-generating associations rather than evidence of chemoprevention and may reflect confounding by indication, treatment selection, immortal-time bias, diabetes severity, competing mortality, or healthcare utilization (26).

Target-trial emulation improves the design transparency of observational studies by specifying eligibility criteria, treatment strategies, time zero, follow-up protocols, and target estimands, but it cannot automatically eliminate unmeasured confounding (25, 27). Subsequent safety studies should pre-specify an active-comparator new-user design, stratification by drug and dose, an appropriate latency period, site-specific outcomes, and competing-risk analyses, and should treat no increased risk and reduced risk as propositions requiring different evidentiary thresholds.

### 4.3 Antitumor potential: from metabolic vulnerability to immune modulation

The largest Article-only BERTopic topic (T0, canagliflozin/cell terms) showed a downward topic-specific trend after accounting for annual Article-corpus size (IRR/year=0.84, BH-adjusted q<0.001). Its size primarily reflects research attention within the indexed literature. Some cancers or models express SGLT2/SLC5A2, and restricting glucose uptake may affect tumor-cell metabolic adaptation (28); canagliflozin can also inhibit mitochondrial complex-I-supported respiration and suppress proliferation in experimental systems (29). In liver-cancer models, canagliflozin was associated with reduced glucose uptake and angiogenic activity (20) and attenuated hepatocarcinogenesis in NASH-related models (30, 31). These findings support testable mechanistic hypotheses (32), while transporter-independent mitochondrial or systemic metabolic actions should also be considered.

On this basis, research has further proposed candidate mechanisms involving stress and growth signaling, cell death, autophagy, angiogenesis, and inflammation (6). These mechanisms help explain connections among studies but are currently better regarded as testable biological hypotheses than as established common pathways of action. Liver-cancer models suggest that canagliflozin may reduce chemoresistance through metabolic reprogramming (33); in a mouse renal-cancer–sunitinib model, dapagliflozin simultaneously improved cardiotoxicity phenotypes and enhanced tumor sensitivity to sunitinib (34). These findings explain the continued activity of mechanistic topics and also indicate that SGLT2 inhibitors should not be regarded as homogeneous exposures; different agents may differ materially in pharmacokinetics, target selectivity, and off-target activity.

Whether exposure levels, target dependence, and model extrapolation can be aligned is a key obstacle to moving from mechanism to clinic. Concentrations required to inhibit tumor growth *in vitro* may exceed clinically achievable free exposures at approved doses, and SGLT2 expression does not itself establish tumor dependence on the transporter. Direct antitumor development therefore requires pharmacokinetic–pharmacodynamic studies matched to clinical exposure, evidence of direct target engagement, comparisons of structurally distinct inhibitors, and biomarker selection; immune mechanisms should be validated in orthotopic and immunocompetent models. In the exploratory evidence map, antitumor/repurposing records remained heterogeneous and were not evidence of established antitumor efficacy (21).

### 4.4 Cardio-oncology

Cardio-oncology currently represents a coherent translational research pathway, but its primary aim is to maintain cardiovascular safety and continuity of cancer treatment rather than to define SGLT2 inhibitors directly as anticancer drugs. Article-only topics included cardio-oncology-related patient/outcome terms and dox/doxorubicin or cisplatin-related terms, presenting a research sequence from mechanisms to clinical outcomes. The benefits of SGLT2 inhibitors in heart failure and kidney outcomes provide a mature background for supportive care (2, 3). Current guidelines emphasize baseline risk stratification, dynamic monitoring, and multidisciplinary management, but evidence for preventive use in oncology remains limited (8).

Existing observational studies provide directionally consistent but still non-causal signals. Among patients treated with anthracyclines, use of SGLT2 inhibitors was associated with fewer cardiac events (35), and fewer clinical events were also observed in patients with cancer therapy-related cardiac dysfunction (36). Real-world comparative research in patients with diabetes and cancer further suggested a lower risk of CTRCD, although treatment selection, baseline cardiovascular risk, and differences in healthcare resources may still result in residual confounding (22). The direction of recent systematic reviews and meta-analyses is consistent with these signals, but their included evidence remains mainly retrospective and cannot replace randomized trials (23, 24, 37, 38).

Prospective evidence remains limited. EMPACARD-PILOT observed that empagliflozin was associated with a lower 6-month incidence of CTRCD in selected patients at high risk during anthracycline treatment, but its size and design are insufficient to replace randomized trials (7). Preclinical research has likewise suggested that empagliflozin can attenuate doxorubicin-related myocardial injury (39). The next step should harmonize definitions of CTRCD and imaging/biomarker endpoints while recording completion of anticancer treatment, cancer outcomes, kidney outcomes, and safety events (40). If the same drug is also proposed to have tumor-sensitizing effects, cardioprotection trials must concurrently demonstrate that it does not compromise antitumor efficacy.

### 4.5 Real-world evidence and emerging clinical-oncology signals

Beyond safety assessment and supportive care, recent contextual studies have addressed disease progression and treatment response. The BERTopic map contains patient/cancer, cancer/sglt2i/dpp4i, and HCC/sglt2i term clusters, but these labels describe research attention rather than pooled clinical effects. Zheng et al. (9) used drug-target Mendelian randomization and observational data to suggest an association with prostate-cancer risk. Genetic instrumental variables represent lifelong target perturbation, whereas clinical cohorts represent short-term drug use and cannot be treated as the same estimand. A small prostate-cancer study reporting PSA response (41) is likewise hypothesis-generating and requires confirmation in larger samples with prespecified time zero and outcomes.

In the context of hormone therapy, SGLT2 inhibitor use was associated with later treatment failure, but overall and cancer-specific survival showed no clear differences (10). Hepatocellular-carcinoma findings remain observational associations with study-specific confounding and follow-up limitations (11, 42), while preclinical models provide mechanistic context (20, 31). Renal-cell-carcinoma findings likewise remain observational associations with study-specific confounding and follow-up limitations (26, 34).

Robust evidence triangulation should combine active-comparator new-user cohorts, negative controls, drug-specific analyses, mechanistic biomarkers, and pre-specified target estimands and, where feasible, proceed to pragmatic prospective studies. A more realistic design would prospectively collect cancer-specific outcomes and treatment tolerance among patients with cancer already receiving SGLT2 inhibitors for diabetes, heart failure, or chronic kidney disease. This would avoid prematurely using these agents as off-label anticancer treatment while testing whether they improve cardiovascular safety, treatment completion, or survival.

### 4.6 Proposed conceptual roadmap and priority research questions

Integrating the bibliometric trajectory, BERTopic results, exploratory evidence map, and the contextual clinical literature discussed above, we propose a five-part conceptual framework linking the evolution of research attention to priorities for clinical translation (**Figure 10**). The first four components summarize major shifts in research focus identified in the literature, whereas the final component represents proposed requirements for future translational validation rather than an empirically observed stage. The framework is therefore not intended as a mandatory linear pathway; the same drug may occupy different positions across cancer types, therapeutic contexts, and research questions. For safety questions, long-term and site-specific risks should be re-estimated using active-comparator new-user designs, latency periods, and competing-risk models; for direct antitumor research, translatable mechanisms should be defined through clinically achievable exposure, direct target engagement, drug- and cancer-type specificity, and biomarker selection; for cardio-oncology supportive care, CTRCD definitions and imaging and biomarker endpoints should be harmonized, with concurrent reporting of antitumor efficacy, treatment completion, kidney outcomes, and safety. At the final clinical-validation stage, studies should test causal effects through pre-registered target-trial emulation, prospective studies, or randomized trials centered on explicit target estimands and patient-relevant endpoints.

**Figure 10:**
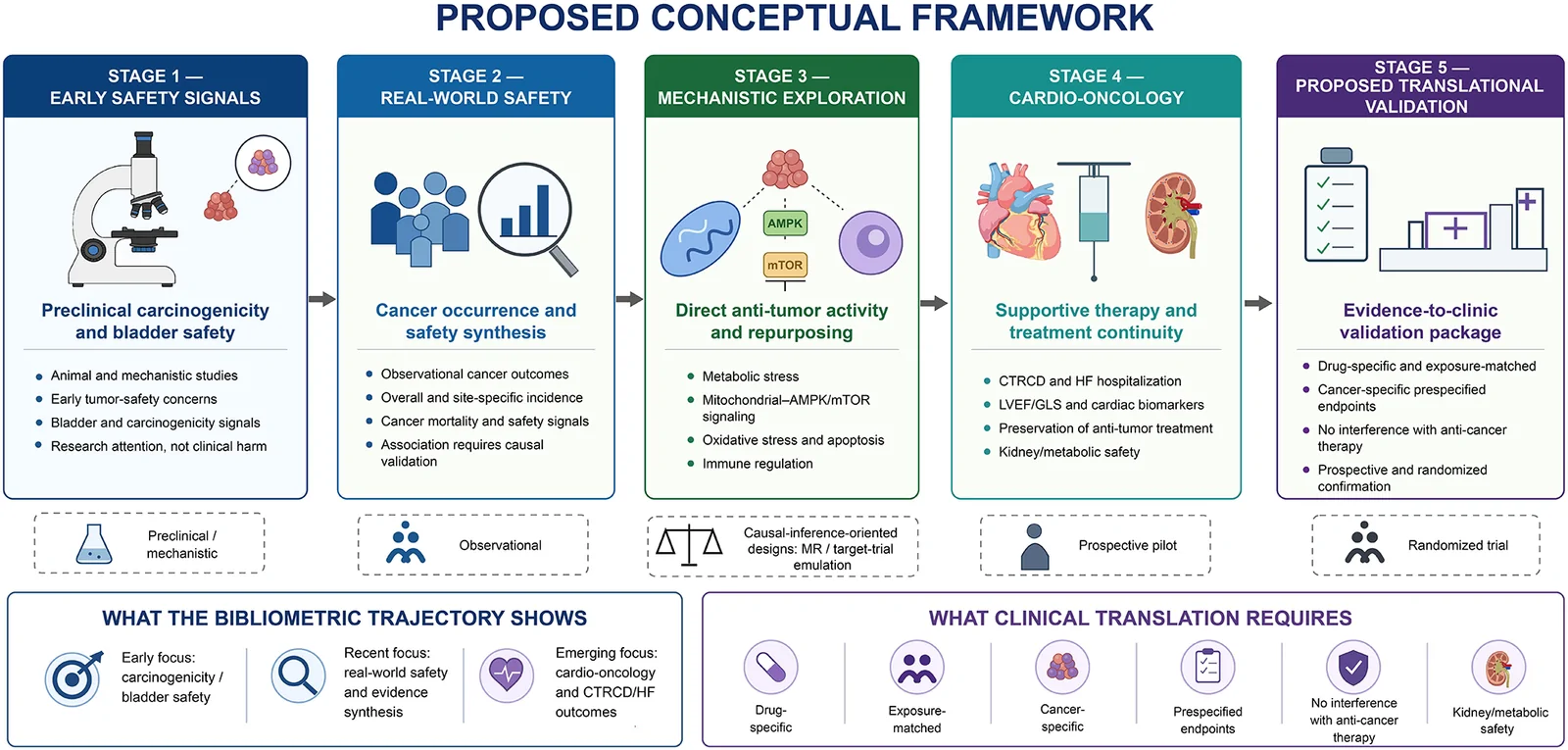
Proposed conceptual framework linking the bibliometric trajectory of SGLT2 inhibitor oncology research to priorities for clinical translation. Stages 1–4 synthesize the major shifts in research attention identified across the bibliometric, semantic, and evidence-mapping analyses, from early safety signals and real-world safety evaluation to mechanistic exploration and cardio-oncology. Stage 5 represents a proposed translational-validation framework rather than an empirically observed stage. Key requirements include drug specificity, clinically achievable exposure, cancer-type specificity, prespecified endpoints, non-interference with anticancer treatment, and kidney/metabolic safety.

### 4.7 Strengths and limitations

The strengths of this study lie primarily in three complementary dimensions. First, the dual-database design balances biomedical coverage with citation structure and explicitly distinguishes the merged corpus of 346 records from the WoSCC citation subset of 283 records and the Article-only topic corpus of 258 records. Second, PubMedBERT-BERTopic provides an independent semantic topic layer, and an Article+Review sensitivity analysis tested the stability of the broad structure. Finally, performance, collaboration, journal knowledge flow, co-citation, bursts, controlled concepts, and the exploratory evidence map were placed within a unified temporal narrative while preserving the distinction between research attention and clinical evidence.

These findings should still be interpreted in light of limitations in study design and data sources. WoSCC and PubMed differ in coverage, update cadence, and field structure. English-language restrictions and search terms may also have led to omissions, and PubMed-unique records could not participate consistently in citation analyses. Citation counts are affected by database indexing and publication age and cannot substitute for methodological quality indicators (12). In addition, network thresholds, synonym normalization, and cluster labels can affect visualizations, and the 2026 literature represents only an incomplete interval through July 26.

BERTopic is likewise model-dependent. UMAP and HDBSCAN parameters can alter the boundaries of small topics, and manual labels are not an external gold standard. The Article-only model yielded 10 topics with 36 outliers, whereas the Article+Review sensitivity model yielded 13 topics; aligned Article assignments showed ARI = 0.634, AMI = 0.698, and mean matched-centroid cosine similarity=0.938. These values indicate substantial, though not perfect, stability after document-type separation. Topic frequencies and topic-specific temporal trends should therefore be interpreted as indicators of research attention within the indexed literature.

## 5 Conclusions

SGLT2 inhibitor oncology research has expanded rapidly since 2021 and has differentiated into parallel tracks of cancer safety, direct antitumor hypotheses, real-world outcomes, and cardio-oncology supportive care. Article-only BERTopic and the Article+Review sensitivity analysis show that the semantic structure is reasonably stable but not identical after Reviews are separated. The bibliometric results map research attention; they do not confirm clinical benefit or exclude rare, site-specific, agent-specific, or latency-related cancer risks. Progress toward clinical translation should therefore require drug-specific, exposure-matched, cancer-specific and patient-relevant prospective or randomized evidence.

## Data Availability

The raw data supporting the conclusions of this article will be made available by the authors, without undue reservation.
